# Vitamin D and Stroke: Effects on Incidence, Severity, and Outcome and the Potential Benefits of Supplementation

**DOI:** 10.3389/fneur.2020.00384

**Published:** 2020-06-10

**Authors:** Keerthi Yarlagadda, Nicholas Ma, Sylvain Doré

**Affiliations:** ^1^Doré Lab, Department of Anesthesiology, Center for Translational Research in Neurodegenerative Disease, McKnight Brain Institute, University of Florida College of Medicine, Gainesville, FL, United States; ^2^Doré Lab, Departments of Neurology, Psychiatry, Pharmaceutics, Psychology, and Neuroscience, McKnight Brain Institute, University of Florida College of Medicine, Gainesville, FL, United States

**Keywords:** vitamin D, ischemic stroke, risk, outcome, supplementation

## Abstract

Vitamin D serum level has been positively associated with improved cardiovascular health, especially with reduction of stroke risk. This systemic review summarizes and synthesizes findings from studies relevant to the relationship between vitamin D and stroke risk, severity, and outcome; potential mechanisms explaining such a relationship; and outcomes from vitamin D supplementation. The literature shows that vitamin D deficiency is a significant risk factor for ischemic stroke, with sun exposure, sex, age, race, diabetes, and genetics playing a role as well. Stroke severity and short- and long-term outcomes also worsen with vitamin D deficiency. The neuroprotective mechanisms by which vitamin D operates to mitigate stroke onset and outcomes have yet to be fully studied, but researchers have proposed several pathways, including promotion of certain neuroprotective growth factors, reduction of arterial pressure through vasodilation, and inhibition of reactive oxygen species. There is some evidence that vitamin D supplementation could lower stroke risk and improve recovery, though outcomes can also be negligible or negative. Although results are mixed and the limitations of vitamin D supplementation merit some caution, vitamin D overall plays a significant role in stroke health. Future research should further develop understanding of the neuroprotective mechanisms of vitamin D and study how supplementation could be administered effectively in stroke treatment.

## Introduction

Stroke is the second leading cause of death worldwide, accounting for over 10% or 5.7 million deaths annually, with the total number of cases predicted to rise over the next few decades (1). *Although stroke mortality has declined to 40–60% in the United States and other developed countries because of strict blood pressure control, the burden of stroke is still rising due to an increase in the older population* *(**1*, *2**)*. Likewise, increased longevity in developing nations has led to rising stroke prevalence in middle-income countries (1, 2).

Vitamin D (VD) is an organic compound consisting of fat-soluble ecosteroids mainly responsible for regulation of calcium and phosphorous levels, among other physiological functions (3, 4). Calcitriol [1,25(OH)2D3] is the active form of VD that binds to the VD receptor

(VDR), dimerizes with retinoid X receptor (RXR), and translocates to the nucleus to bind to VD response elements (VDRE) (5). VD is measured by levels of a metabolically inactive precursor, 25-hydroxyvitamin D_3_hydroxy VD [25(OH)D_3_D3], since the serum concentration of 1,25(OH)_2_D_3_ (≤ 100 pM) is significantly lower than 25(OH)D_3_D3 (≤ 100 nM) (6). Optimal 25(OH)D_3_ levels are at least 30 ng/ml (75 nmol/L) 25(OH)D_3_(3), with levels below 12 ng/mL (30 nmol/L) considered deficient by the Institute of Medicine (7). 25(OH)D_3_ deficiency can cause bone demineralization and is associated with obesity, diabetes, hypertension, and cancer (3, 4, 8). 25(OH)D_3_ levels have been associated with regulating cardiac myocyte, systolic blood pressures, glycemic control, vascular function, high-density cholesterol, and metabolic syndrome, which all influence cerebrovascular and cardiovascular events (3, 4, 8–15). 25(OH)D_3_-deficient patients are recommended to orally consume at least 50,000 IU (1,250 mcg) of ergocalciferol, or VD2, once per week or more for 6–8 weeks, followed by 800–1,000 IU (20–25 mcg) daily (16).

Researchers are increasingly focusing more on the relationship between VD and stroke. Literature reviews have identified 25(OH)D_3_ deficiency as an independent risk factor for stroke, highlighting the potential for using supplementation as a treatment for stroke (17–19). This review paper seeks to update the literature on VD's association with stroke while also highlighting the promises and limitations of supplementation in stroke treatment and prevention.

## Vascular Outcomes From Experimental Data

Although 1,25(OH)_2_D_3_'s mechanism against ischemic stroke has yet to be fully understood, several neuroprotective mechanisms have been proposed (20–24). 1,25(OH)_2_D_3_ can promote the expression of insulin-like growth factor 1 (IGF-1), which has neuroprotective capabilities that help combat axon and dendrite degeneration (20, 23), as well as antithrombotic capabilities through activation of plasminogen (20, 21). In light of this mechanism, diabetes may serve more as a causal link between VD and stroke than as a confounding variable, as suggested in recent findings (12, 25).

1,25(OH)_2_D_3_ could also induce vasodilation, relieve arterial pressure, and improve post stroke blood flow to neurons by potentiation of nitric oxide synthase (NOS) (20, 23). These benefits can especially be seen in 1,25(OH)_2_D_3_'s anti-inflammatory effects and attenuation of cerebral vasospasm in hemorrhagic stroke development, as measured by vessel diameter and endothelial function of the basilar artery (26). Anti-inflammation of myeloid and endothelial cells is believed to be the result of 1,25(OH)_2_D_3_ induction of stromal cell-derived factor 1α (SDF1α), vascular endothelial growth factor (VEGF), and endothelial NOS (26). In mice, VD inhibits renin-angiotensin, a vasoconstrictor, and thus can help reduce blood pressure and hinder the onset of cardiovascular diseases (22). Therefore, lower 25(OH)D_3_ levels may lead to vascular stiffness, supported by evidence of higher abnormalities in right and left brachioarterial pulse wave velocities (PWV) and carotid-femoral PWV among 25(OH)D_3_-deficient and -insufficient patients with coronary artery disease (27). Narrower and stiffer vessels due to 25(OH)D_3_ deficiency could therefore increase the risk of occlusion; indeed, low 25(OH)D_3_ levels in patients with ischemic stroke have been strongly linked to deep venous thrombosis development (28). 1,25(OH)_2_D_3_, as an antioxidant, also inhibits reactive oxygen species production to prevent blood brain barrier (BBB) dysfunction in cerebral endothelial cells after an ischemic stroke in a mouse stroke model (24). In a male rat model, deficiency in 25(OH)D_3_ has been found to significantly decrease expression of tight-junction proteins occludin and claudin-5 after ischemic stroke, therefore leading to greater BBB dysfunction (29). 25(OH)D_3_ deficiency will not only reduce neuroprotection, but also cause conditions linked to cardiovascular disease. For instance, hyperparathyroidism, triggered by 25(OH)D_3_ deficiency to counteract low serum calcium, has been linked to inflammation and cardiac hypertrophy (21). Furthermore, lower 25(OH)D_3_ levels in patients with stroke is associated with more severe white matter lesions, enlarged perivascular spaces, and MRI-detected cerebral small-vessel disease burden (30).

## Functional Outcomes From Observational Studies

25(OH)D_3_ deficiency has been linked to greater stroke severity and negative post stroke outcomes (20–22, 31–35). Lower serum 25(OH)D_3_ levels in patients with stroke are independently associated with higher infarct volumes, although causality has yet to be determined (20–22, 36). Overall stroke severity, assessed using the National Institutes of Health Stroke Scale, was worse in 25(OH)D_3_-deficient patients with stroke, whereas patients with sufficient to optimal VD levels had lower scores on that scale, or less severe strokes, on average (20–22, 31, 32, 37). Short-term post stroke outcome, measured by the modified Rankin Scale (mRS) at patient discharge, was similarly poorer among 25(OH)D_3_-deficient patients with stroke (20, 31–34, 36). 25(OH)D_3_-deficient patients also had higher mRS scores 3 months post stroke, reflecting relatively worse longer-term outcomes (20, 31, 32, 34, 37). A significant nonlinear relationship between 25(OH)D_3_ and Barthel Index scores has also been found at 3 months, with the strongest association up to 16 ng/ml 25(OH)D_3_ before the effects of higher 25(OH)D_3_ diminish (38). Likewise, cognitive impairment at 1 month after ischemic stroke, assessed by the Mini-Mental State Examination and adjusted for risk factors, was also significantly higher among 25(OH)D_3_-deficiency patients, although not significantly different between 25(OH)D_3_-sufficient and -insufficient patients (39). Regarding longer-term outcomes, only one study with a small sample of 50 patients, found no significant correlation between mRS scores and 25(OH)D_3_ after 6 months (35).

Lower 25(OH)D_3_ in patients with ischemic stroke has also been correlated with poorer scores for other metrics assessing mental and physical post stroke outcome. Such metrics include the Functional Independence Measurements of Motor and Cognitive Function, Functional Ambulatory Scale, and the Mini-Mental State Examination; however, there was no such correlation for haemorrhagic stroke (23). Overall cardiovascular disease mortality has been correlated with lower 25(OH)D_3_ levels (22). There is an inverse relationship between 25(OH)D_3_ levels and 1-year mortality for ischemic stroke patients younger than age 75, after adjusting for other risk factors, although the association between death and 25(OH)D_3_ deficiency was no longer observed for patients older than 75 years (40). Ischemic stroke recurrence within at least 3 months has also been found to be negatively correlated with serum 25(OH)D_3_ levels (21). The negative association between 25(OH)D_3_ levels and stroke recurrence, as well as mortality, continues for at least up to 24 months (41). Prospective population health studies observing dietary intake have also noted that dietary intake of VD in middle-aged to senior populations was associated with reduced risk of stroke incidence and mortality (42, 43). The negative association between 25(OH)D_3_ levels and stroke recurrence, as well as mortality, continues for at least up to 24 months (41).

VD deficiency (VDD), with serum levels lower than 20 ng/ml (50 nmol/L) 25(OH)D_3_, can cause bone demineralization and is associated with obesity, diabetes, hypertension, and cancer (3, 4, 8). VD levels have been associated with regulating cardiac myocyte, systolic blood pressures, glycemic control, vascular function, high-density cholesterol, and metabolic syndrome, which all influence cerebrovascular and cardiovascular events (3, 4, 8–15, 44). VDD patients are recommended to orally consume at least 50,000 IU (1,250 mcg) of VD once per week or more for 6–8 weeks, followed by 800–1,000 IU (20–25 mcg) daily.

Researchers are increasingly focusing more on the relationship between VD and stroke. Literature reviews have identified VDD as an independent risk factor for stroke, highlighting the potential for using supplementation as a treatment for stroke (17–19). This review paper seeks to update the literature on VD's association with stroke while also highlighting the promises and limitations of supplementation in stroke treatment and prevention.

## Variables Explored in Observational Studies Behind Correlation Between VD And Stroke

### Sun Exposure

VDVD synthesis requires adequate sunlight exposure, little of which is attained in regions above and below 33° latitude during the winter, and only occurring at peak zenith angles of the sun (45). Nevertheless, a meta-analysis indicates no significant correlation on a global scale (46). There are other variables to consider when explaining 25(OH)D_3_ levels. *Population residing in the extreme northern and southern regions where sun exposure is inadequate have a low level of VD. Air particulate pollution can also reduce sun exposure and also effects D synthesis in the body* (47). Nonetheless, 25(OH)D_3_ deficiency is prevalent worldwide across different latitudes (48); the regions with the greatest deficiency include the Middle East, Asia and Northern Europe. Nonetheless, VD deficiency is prevalent worldwide across different latitudes (48–50); the regions with the greatest VD deficiency include the Middle East, Asia, and Northern Europe (47). In the United States alone, an estimated 40% of the population has deficient 25(OH)D_3_ levels (51).

There is little research on the seasonal and latitudinal influences on exposure to sunlight and stroke incidence due to 25(OH)D_3_ deficiency. Some evidence suggests that 25(OH)D_3_ deficiency due to low sun exposure at higher altitudes has contributed to greater risk for cardiovascular disease through hypertension (52–54). Similarly, variation in 25(OH)D_3_ between seasons of low sun exposure (October to March) and high sun exposure (April–September) have been found to be an accurate predictor of coronary artery disease (10). In Greece, the peak incidence of ischemic stroke occurs in spring (8.4% above average), whereas summers have the lowest incidence of stroke (10.4% below average); however, there were no significant seasonal differences in intracerebral hemorrhage, subarachnoid hemorrhage, and transient ischemic attack incidence.

There is little research on the seasonal and latitudinal influences on exposure to sunlight and stroke incidence due to VDD. Some evidence suggests that VDD due to low sun exposure at higher altitudes has contributed to greater risk for cardiovascular disease through hypertension (52, 53, 55, 56). Similarly, variation in 25(OH)D_3_ between seasons of low sun exposure (October to March) and high sun exposure (April–September) have been found to be an accurate predictor of coronary artery disease (46, 57). In Greece, the peak incidence of ischemic stroke occurs in spring (8.4% above average), whereas summers have the lowest incidence of stroke (10.4% below average); however, there were no significant seasonal differences in intracerebral hemorrhage, subarachnoid hemorrhage, and transient ischemic attack incidence (56). Additionally, below-median sun exposure for a year has been found to significantly increase the risk of stroke incidence [hazard ratio (HR) 1.61] (58). Many stroke studies have already adjusted for sun exposure due to season and/or latitude among many other variables influencing 25(OH)D_3_ levels (8, 12–14, 59–61), so the impact of sun exposure alone has yet to be thoroughly studied.

### Sex

Several studies have focused on sex-specific relationships between VD and stroke (11, 60, 62–64). A female retrospective cohort study from 1968 to 2001 involving middle-aged Swedish women born in Gothenburg found that women with <50 nmol/L in their blood not only had a higher risk for cardiovascular disease (HR 1.29), but also a significant increase in stroke risk (HR 3.30) and morbidity (HR 1.96) for the first 17 years of the study, after accounting for all confounding variables (62). Inevitably, though, the relative risks converged for all women, indicating that the physiological risks of 25(OH)D_3_ deficiency peak around 50 to 70 years of age and eventually subside. Many stroke studies have already adjusted for sun exposure due to season and/or latitude among many other variables influencing VD levels (8, 12, 13, 59–61, 65, 66), so the impact of sun exposure alone has yet to be thoroughly studied.

A similar case-cohort study examined 928 female nurses, half of whom were patients with stroke and the other half of whom were the healthy control, from the Nurses' Health Study from 1976 to 2006 (60). It was observed that the women in the lowest tertile of 25(OH)D_3_ levels (9.2–45.7 nmol/L) experienced a moderate risk increase for stroke [odds ratio (OR) 1.49], suggesting a modest association between 25(OH)D_3_ deficiency and stroke risk (60, 66). Inevitably, though, the relative risks converged for all women, indicating that the physiological risks of VDD peak around 50 to 70 years of age and eventually subside (25, 62). A similar case-cohort study examined 928 female nurses, half of whom were patients with stroke and the other half of whom were the healthy control, from the Nurses' Health Study from 1976 to 2006 (60). Another study examined both males and females enrolled in the National Health and Nutrition Examination Survey from 2001 to 2006 and found that higher 25(OH)D_3_ levels reduced stroke risk the most for women ages 20 to 50 (OR 0.26) (63), supporting the conclusions of the Swedish cohort study regarding the importance of VD for middle-aged women (62).

In contrast, studies focusing on men, such as a prospective cohort study associated with the Osteoporotic Fractures in Men, found no significant association between 25(OH)D_3_ levels and risk for cardiovascular or congenital heart disease, although 25(OH)D_3_ deficiency increased risk for a cerebrovascular or stroke event (HR 1.70) (11). Another prospective cohort study involving middle-aged men, part of the Honolulu Heart Program, found that Japanese men in the lowest quartile of VD intake (0–44.8 IU) had a slight increase in risk for all stroke events (HR 1.22) which includes risk for thromboembolism or ischemic stroke (HR 1.27). There was no significant association with hemorrhagic stroke, and the risk for stroke in 25(OH)D_3_-deficient middle-aged men is far more modest compared with women (64). The overall research demonstrates that stroke risk for middle-aged 25(OH)D_3_-deficient women is far more significant and severe than it is for middle-aged men (11, 60, 62–64).

### Race

The influence of race on the relationship between VD and stroke is unclear. Relatively few studies have focused exclusively on racial differences in the relationship between VD levels and stroke (67–69). A prospective cohort study, part of the Third National Health and Nutrition Survey from 1988 to 1994, found that while black Americans tended to have significantly lower 25(OH)D_3_ levels than white Americans, the association between severe VDD (<15 ng/ml 25(OH)D_3_) and stroke was only significant in whites (HR 2.13) but not in blacks (HR 0.93) (67). However, a more recent retrospective cohort study examining a larger population of 29,653 patients, half-white and half-black, from 2003 to 2007, revealed that VDD individuals (<20 ng/ml 25(OH)D_3_) were more likely than individuals with optimal VD (>30 ng/ml 25(OH)D_3_) to be stroke victims (HR 1.85), with the relationship decreasing for VD-insufficient individuals (20–30 ng/ml 25(OH)D_3_; HR 1.33) (68). Evidence from the Atherosclerosis Risk in Communities study performed across the United States similarly found no significant difference between blacks and whites in the association between 25(OH)D_3_ levels and stroke risk (69). Despite prior contrary evidence (67), recent findings suggest no racial differences in the relationship between VD and stroke risk (68, 69).

There was no significant difference in this inverse association of 25(OH)D_3_ and stroke risk between races (68). Evidence from the Atherosclerosis Risk in Communities study performed across the United States similarly found no significant difference between blacks and whites in the association between 25(OH)D_3_ levels and stroke risk (69). Despite prior contrary evidence (64, 69), recent findings suggest no racial differences in the relationship between VD and stroke risk (68, 69).

### Diabetes

Among non-diabetic patients, there is evidence of a positive correlation between 25(OH)D_3_ deficiency and stroke risk (25, 70). A Chinese randomized single-blind clinical trial found that 25(OH)D_3_ deficiency in normoglycemic or non-diabetic patients could increase stroke risk (HR 1.58) (25). Similarly, a Chinese retrospective cohort study found that middle-aged non-diabetic patients with ischemic stroke were over three times more likely to have worse stroke outcomes (OR 3.20) and had almost four times the risk of mortality with 25(OH)D_3_ deficiency (OR 3.90) (70). However, there is some debate about stroke risk from 25(OH)D_3_ deficiency among diabetic patients, with some studies suggesting that diabetes is a confounding variable (12, 25) and another study finding otherwise. There is also debate about stroke risk from VDD among diabetic patients, with some studies suggesting that diabetes is a confounding variable (12, 25) and another study finding otherwise (71). For instance, 25(OH)D_3_ deficiency was associated with a reduced stroke risk among normoglycemic patients (HR 1.58), but this relationship was not observed in hyperglycemic patients (25). An American 7-year prospective cohort study similarly found that while 25(OH)D_3_ deficiency is moderately associated with stroke in 65–67-year-old patients (HR 1.30), the risk significantly diminishes after adjusting for diabetes (HR 1.11) (12). A German retrospective cohort study of 1,108 diabetic patients, however, still found 25(OH)D_3_ deficiency to be a significant risk factor for stroke (HR 2.58), as well as for sudden cardiac death (HR 2.34) (71).

An American 7-year prospective cohort study similarly found that while VDD is moderately associated with stroke in 65–67-year-old patients (HR 1.30), the risk significantly diminishes after adjusting for diabetes (HR 1.11) (11). A German retrospective cohort study of 1,108 diabetic patients, however, still found VDD to be a significant risk factor for stroke (HR 2.58), as well as for sudden cardiac death (HR 2.34) (71).

### Genetics

Recent evidence suggests an underlying genetic role in the association between 25(OH)D_3_ deficiency and stroke (69, 72–74). 25(OH)D_3_ levels are regulated by carrier VD binding proteins (DBP) (5), and genotypes for high DBP single-nucleotide polymorphism (SNP), particularly the G allele of rs7041 and A allele of rs4588, are linked with low 25(OH)D_3_ levels (69, 72). Genetic predisposition toward high DBP SNP, particularly with genotypes rs7041 TG/GG (HR 1.29) and rs4588 CA/AA (HR 1.37), is therefore a significant factor in stroke risk through the reduction of 25(OH)D_3_ serum levels (69). Other genetic variants associated with serum 25(OH)D_3_ have little significant causal relationship with ischemic stroke (73, 74). Such variants include DBP SNPs (rs1155563, rs2282679, rs12785878, and rs3829251), which are linked to below-average 25(OH)D_3_ and also have no significant influence on other cardiovascular diseases such as myocardial infarction (73). Similarly, mutations in the DHCR7 and CYP2R1 genes, which can genetically predetermine low 25(OH)D_3_ levels, have a slight association with hypertension (OR 1.02), but no association with ischemic stroke (OR 0.98) (74). Therefore, 25(OH)D_3_ deficiency and stroke risk does have a genetic component, with variants rs7041 TG/GG and rs4588 CA/AA significantly linked to ischemic stroke risk (69, 72).

## Randomized Controlled Trials of VD Supplementation

The few randomized controlled trial studies examining the efficacy of VD supplementation suggest that VD intake could also improve stroke outcome and cardiovascular function. A non-blinded randomized controlled trial on 25(OH)D_3_-deficient and -insufficient stroke patients tested the effects of administering single doses of 600,000 IU of Cholecalciferol Intramuscular injections (75). The experimental group's mean functional outcomes at 3 months improved by 6.39 points on the Scandinavian Stroke Scale, whereas the control group improved by only 2.5 points (75). The results of this study, however, are questionable due to the non-blinded nature of the trial. Another randomized controlled trial tested VD-calcium supplementation; after 6 months, 25(OH)D_3_-deficient patients with supplementation had a decreased mortality risk (HR 0.26) compared with the control group and were more likely to attain a good mRS functional outcome (OR 1.90) (76). However, at doses of 2,000 IU per day for 5 years, as tested in a nationwide randomized, placebo-controlled trial, VD supplementation failed to reduce the incidence of cardiovascular events and mortality (77).

A significant body of randomized controlled trials has examined vascular outcomes from VD treatment. A randomized, placebo-controlled trial found that for overweight/obese 25(OH)D_3_-deficient patients, higher dosage supplementation was associated with decreased mean carotid-femoral PWV and carotid-radial PWV, whereas the placebo group had significantly higher arterial pressure (78). This reduction in PWV is consistent with the proposed protective mechanism of VD against cardiovascular disease via arterial pressure reduction (20, 23). Large single doses of VD show mixed improvement in flow-mediated dilation (FMD). One clinical controlled trial that administered a monthly 300,000 IU VD supplement to 25(OH)D_3_-deficient patients (<25 nmol/L) found significant improvements in FMD and thiobarbituric acid-reactive substances (TBARS) after treatment; however, the study was not randomized and placebo-controlled (79). When patients were administered single doses of VD2 (100,000 IU) to patients with stroke history in a randomized, placebo-controlled, double-blind trial, FMD improved from 3.7 to 6.9% after 8 weeks (80). Furthermore, large single dosages of VD supplementation could assist 25(OH)D_3_-deficient patients with comorbidities. A double-blind, parallel group, randomized, placebo-controlled trial that administered a one-time dose of 100,000 IU VD2 on 25(OH)D_3_-deficient, Type 2 diabetic patients found improvement in FMD compared with the placebo (81). Children between 3 and 20 years old with chronic kidney disease and 25(OH)D_3_ deficiency likewise had a significant increase in FMD. Endothelium-independent FMD, a measure of arterial stiffness, after cholecalciferol supplementation significantly decreased in an interventional study (82).

However, as noted previously, a relatively lower dose of VD administered frequently does not necessarily lead to improved endothelial outcome. The daily administration of 2,500 IU of VD3 for 4 months to 25(OH)D_3_-deficient women resulted in no FMD and PWV differences between experimental and placebo groups (83). Likewise, a higher daily dosage of 5,000 IU for 12 weeks for Type 2 diabetic patients, who have benefitted from large, single VD doses (81), led to few significant improvements in FMD, circulating endothelial progenitor cells, or PWV (84). Daily statin use, which increases serum 25(OH)D_3_ levels similarly to VD supplements, can reduce carotid intima-media thickness and increase circulation of endothelial progenitor cells but has failed to significantly improve FMD (85).

Furthermore, VD and calcium supplementation may have negative (86) to negligible (87–89) effects. Recent evidence suggests an underlying genetic role in the association between VDD and stroke (20, 31–33, 69, 72–74). 25(OH)D_3_ levels are regulated by carrier VD binding proteins (DBP) (5), and genotypes for high DBP single-nucleotide polymorphism (SNP), particularly the G allele of rs7041 and A allele of rs4588, are linked with low 25(OH)D_3_ levels (69, 72). Genetic predisposition toward high DBP SNP, particularly with genotypes rs7041 TG/GG (HR 1.29) and rs4588 CA/AA (HR 1.37), is therefore a significant factor in stroke risk through the reduction of 25(OH)D_3_ serum levels (69). Other genetic variants associated with serum 25(OH)D_3_ have little significant causal relationship with ischemic stroke (73, 74). Such variants include DBP SNPs (rs1155563, rs2282679, rs12785878, and rs3829251), which are linked to below average 25(OH)D_3_ and also have no significant influence on other cardiovascular diseases such as myocardial infarction (73). Similarly, mutations in the DHCR7 and CYP2R1 genes, which can genetically predetermine low 25(OH)D_3_ levels, have a slight association with hypertension (OR 1.02), but no association with ischemic stroke (OR 0.98) (74). Therefore, VDD and stroke risk does have a genetic component, with variants rs7041 TG/GG and rs4588 CA/AA significantly linked to ischemic stroke risk (69, 72).

### Functional Outcomes and VD on Brain Protection

**Figure 1:**
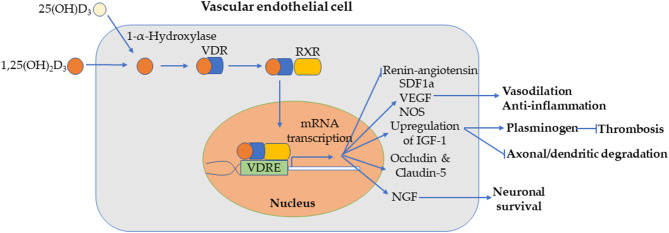
Combination of various proposed mechanisms in endothelial cells for vitamin D prevention/mitigation of ischemic stroke. 1,25(OH)_2_D_3_ [or 25(OH)D_3_D3, which is converted to of 1,25(OH)_2_D_3_ via 1-α-Hydroxylase] diffuses through the endothelial cell membrane, binds to vitamin D receptor (VDR), dimerizes with RXR, and translocates into the nucleus. The complex binds to VDRE for transcription of genes resulting in inhibition of renin-angiotensin and activation of SDF1a, VEGF, and NOS pathways for vasodilation and anti-inflammation; upregulation of IGF-1 (and thus neuroprotection of axon and dendrites and thrombolysis via plasminogen); expression of blood brain barrier (BBB) tight-junction proteins occludin and claudin-5; upregulation of nerve growth factor (NGF), which supports neuronal growth, maintenance, and survival.

Prospective population health studies observing dietary intake have previously noted that dietary intake of VD in middle-aged to senior populations has reduced the risk of stroke incidence and mortality (42, 43). The few randomized controlled trials examining the efficacy of VD supplementation suggest that VD intake could also improve stroke outcome and cardiovascular function (Figure 1).

Furthermore, VD and calcium supplementation may have negative (86) to negligible (87, 89, 90) effects. A meta-analysis combining data from the Women's Health Initiative and eight other studies, encompassing 28,072 patients, found that calcium supplementation, with or without VD, could modestly increase the risk of myocardial infarction and stroke (86). It is theorized that the increased serum calcium levels from dietary intake could lead to carotid artery plaque thickness and aortic calcification (86). However, an observational cohort study on VD and calcium supplementation found no increase in the incidence of myocardial infarction, stroke, or mortality for women who have consistently received supplementation after 2 years, relative to women who received minimal supplementation (87). A recent nested case-control study from the American Heart Association further clarifies prior findings (86, 87) by noting that high daily calcium supplementation (≥1,000 mg) may lead to an increased risk of ischemic stroke, but the combination of VD supplementation negates such risk (89). It is likely that VD absorption of calcium prevents high serum calcium (3, 4) and the corresponding artery plaque thickness and aortic calcification.

Meta-analyses on VD-only supplementation studies have similarly found mixed results (81, 82), as have meta-analyses of randomized controlled trials studying VD-alone supplementation (91–94). One found that VD supplementation did not significantly improve FMD overall, but supplementation was found to be most effective in studies that lasted <16 weeks (suggesting only short-term benefits) and with patients with systolic blood pressure >140 mmHg and diastolic blood pressure <80 mmHg (91). Likewise, a smaller meta-analysis suggested that VD supplementation may improve FMD, although the *p*-value (0.054) was too high to be significant; the authors believed that more studies were required for significance (92). A systematic review and individual participant meta-analysis found no significant impact from VD3 supplementation on FMD and PWV, although higher doses of supplementation can create a slightly greater treatment effect on FMD, along with modest improvement for microvascular function (93). These results were supported by a similar systematic review and meta-analysis of randomized controlled trials, which found improvement in FMD, although not in PWV and augmentation index, for metabolic syndrome patients receiving VD supplementation (94).

## Discussion

**Figure 2:**
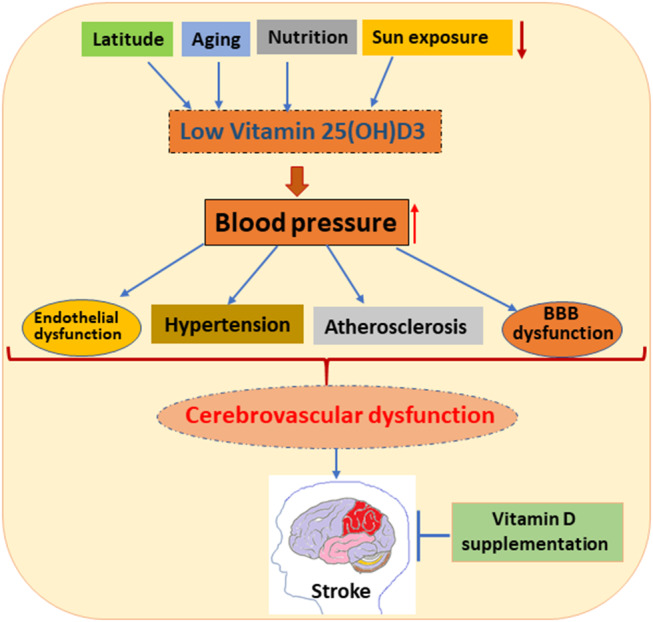
Schematic diagram represents the effects of low 25(OH)D_3_ on various vascular-related functions such as endothelial function, hypertension, atherosclerosis, and blood brain barrier (BBB) dysfunction. Various factors, such as latitude, aging, nutrition, and sun exposure cause VDD, which affects blood pressure. Elevated blood pressure causes vascular impairments, leading to cerebrovascular impairment and thus stroke in patients. Many clinical and preclinical studies show that VD supplementation improves stroke outcome.

The body of literature examined mostly consists of retrospective or prospective studies analyzing the relative risk of ischemic stroke or cardiovascular disease in general from 25(OH)D_3_ levels. Relatively few randomized controlled trials directly investigate the effects of VD supplementation, and they have small sample sizes. Other supplemental randomized controlled studies that examine significantly larger populations tend to focus on calcium supplementation. Further randomized controlled trials strictly examining VD supplementation with larger sample sizes would provide clearer insight on the clinical implications for stroke treatment and prevention (Figure 2, Tables 1, 2).

**Table 1:** Summary of vitamin D benefits, mechanisms, supplementation.

**Associations with ischemic stroke**	**Proposed mechanisms**	**Recommended supplementation**
• Lowered risk • Reduced severity • Improvement in mental and physical outcome • Reduced recurrence and mortality	• NO pathway °Vasodilation °Inhibit renin-angiotensin (vasoconstriction) • Maintains BBB integrity • IGF-1 and other growth factor pathways °Prevents axon/dendrite degeneration °Antithrombosis (plasminogen) • Others	• Adults: 100,000–300,000 IU (2,500–7,500 mcg) monthly^*^ • ≥30 ng/ml serum 25(OH)D_3_ optimal

* *Values based on positive results for single large doses (81, 82)*.

**Table 2:** Effects of low vitamin D on stroke outcomes.

**Patients with vitamin D deficiency**	**Comorbid conditions/ secondary outcomes**	**Cardiovascular risk /Stroke incidence**
Patients with low serum 25-(OH)D	Metabolic syndrome	Increased cardiovascular risk in patients (4, 95)
Children with 25(OH)D concentrations <30 ng/mL	Severe obesity	Increased cardiovascular risk (8)
Low plasma levels calcidiol (vitamin D metabolite)	Sun exposure, coronary artery disease, and hypertension	Development of acute ischemic events (acute coronary syndrome, stroke, or transient ischemic attack) (8)
Vitamin D deficiency with serum 25(OH) vitamin D levels <20 ng/mL	Poor health behaviors, comorbid health conditions, and potential biological mediators	Higher risk of cerebrovascular and cardiovascular events such as heart failure, myocardial infarction, stroke, or cardiovascular death (11, 12, 96)
25(OH)D deficiency [defined as serum 25(OH)D levels <20 ng/mL]	Smoking status, hypertension, diabetes, elevated low-density lipoprotein cholesterol, hypertriglyceridemia, low high-density lipoprotein cholesterol, chronic kidney disease	Increased risk of cardiovascular disease (13)
25-dihydroxyvitamin D (25-OH D) levels 25-OH D deficiency (<15 ng/mL, <10 ng/mL)	n/a	Increase in cardiovascular risk (15)
Low levels of 25-hydroxyvitamin D (25[OH]D)	Cardiovascular, musculoskeletal, infectious, autoimmune, and malignant diseases	Cerebrovascular disease and stroke (17)
Low 25(OH)VitD	Air pollution and low sunlight exposure along with severe malnutrition	High risk of cardiovascular disease and stroke (18, 47, 58)
Low vitamin D status (Meta-analysis)	n/a	Increased risk of ischemic stroke not hemorrhagic stroke (19)
25(OH)D concentrations deficient (<10 ng/mL) and insufficient (10–20 ng/mL)	Adverse effects on neurocognitive health and stroke (with and without dementia symptoms)	Cerebrovascular disease and Cardiovascular disease (CVD) stroke (57, 97)
Low 25-hydroxyvitamin D levels (25[OH]D)	Diabetes mellitus, hypertension, and cancer	CVD with 26% increased rate of all-cause mortality (65)
Low serum 25-dihydroxyvitamin D levels	Musculoskeletal health	Acute stroke (59, 59)
Low 25-hydroxyvitamin D (25[OH]D) (<12 ng/mL)	Elevated cardiovascular disease risk	29% higher CVD risk and 3.3-fold elevated risk of ischemic stroke while higher vitamin D levels were significantly associated with reduced risk of stroke (60, 66)
Low levels of 25(OH)D and 1,25(OH)2D	History of previous cerebrovascular disease events	Increased ischemic and hemorrhagic strokes (61)
Low dietary vitamin D intake or serum 25-hydroxyvitamin D deficiency	Age, calories, body mass index, hypertension, diabetes, smoking, physical activity, serum cholesterol, alcohol intake and low high-density lipoprotein cholesterol	Increased all stroke, thromboembolic ischemic stroke (25, 64)
Deficiency of 25-hydroxyvitamin D	Cardiovascular disease events and mortality	The risk of fatal stroke was greater in blacks compared with whites (67).
Low 25-hydroxyvitamin D concentrations	There were no statistically significant differences in the association of stroke in black vs. white participants	Increased risk of stroke in patients with 25-hydroxyvitamin D <20 ng/mL (68)
Low serum 25-hydroxyvitamin D [25(OH)D]	Atherosclerosis Risk in Communities	The lowest quintile of 25(OH)D (<17.2 ng/ml) was associated with higher stroke risk (69)
Non-diabetic with vitamin 25-hydroxyvitamin D (25(OH) D) deficiency	n/a	Increased poor functional outcome events in Chinese non-diabetic stroke individuals (70)
Low 25-hydroxyvitamin D [25(OH)D]	Hemodialysis, diabetes	Severe vitamin D deficiency was strongly associated with stroke, cardiovascular events, and mortality (71, 98)
Low serum 25(OH)D	Multivariable analyses showed that the risk for a poor 90-day outcome doubled with each 10-ng/mL decrease in serum 25(OH)D	Low levels associated with large volume infarcts Higher serum 25(OH)D concentration was associated with smaller infarct volumes (20)
Low serum 25(OH)D level. The mean 25(OH)D level was 47.2 ± 31.7 nmol/l, and most patients met vitamin D deficient status (<50 nmol/l)	Vascular risk factors	Increased stroke severity in patients (20, 31)
Low serum 25-Hydroxyvitamin D (25(OH)D)	Intravenous thrombolysis	Worse functional acute ischemic stroke outcomes (32)
The mean level of 25(OH)D was significantly lower in the chronic group than in the subacute group (12.3 vs. 16.3 ng/mL.	Patients with a history of total parenteral nutrition had lower 25(OH)D levels than subjects who had enteral nutrition	Onset of stroke (33)
Low concentrations of plasma 25-hydroxyvitamin D (25(OH)D) and genetic variants in DHCR7/ CYP2R1	Cardiovascular disease (CVD) risk, myocardial infarction, high blood pressure, hypertension, and	Increased risk of ischemic stroke (73, 74)
Low serum levels of 25(OH) D	Increased risk of cardiovascular disease	Levels were observed to be prognostic markers of cardiovascular disease and all-cause mortality in Chinese patients with ischemic stroke or risk of recurrent stroke (21, 22)
25(OH)D levels	High inflammatory markers	Poor short-term outcome in acute ischemic stroke patients as indicated by modified Rankin scale (36)
Insufficiency of 25(OH) vitamin D was observed after birth in 70% of infants	Lower circulating anti-inflammatory IL-17E	Higher risk of hypoxic-ischemic encephalopathy (99)
25(OH)D deficiency	Hypertension	Stroke severity was worse with National Institutes of Health Stroke Scale (NIHSS) score (37)
Vitamin D deficiency	Cognitive decline was observed	Increased acute ischemic stroke occurrence (39)
Decreased levels of 25-hydroxyvitamin D (25-OH-D)	Cardiovascular risk	Ischemic stroke with increased risk of mortality (100)
Serum 25-hydroxyvitamin D [25(OH)D] levels	Early neurological deterioration	Higher risk of acute ischemic stroke (101)
mean 25(OH)D level was lower <25.7 nmol/l	Age <75 years	Higher mortality in stroke (40)
Lower serum levels of 25(OH) D. (24-month follow-up study in China involving 220 stroke population)	n/a	Patients had recurrence of ischemic stroke with high mortality (41).
High 25(OH)D levels	Improve cognitive function	Stroke patients showed improved neurological function (23)
Vitamin D deficiency	Increase in arterial stiffness, widening of pulse pressure, atherosclerosis, cardiovascular morbidity and mortality	Increased risk of stroke occurrence (27)
Low serum 25(OH)D levels	Deep vein thrombosis (DVT)	Ischemic stroke (28)
Low median 25(OH)D level i.e., 39.2 nmol/L	Cerebral small vessel disease (cSVD)	Minor ischemic stroke or transient ischemic attack were included (30)
386 patients, with low serum 25(OH)D levels	n/a	Increased incidence of stroke (102)
Low intake of vitamin D and certain flavonoids	Age, gender, smoking and functional capacity	Increased acute myocardial infarction and stroke (42)
Dietary vitamin D intake (965 to 970 person-years on follow-up found 1,514 stroke and 702 coronary heart disease patients)	Stroke, intraparenchymal hemorrhage, coronary heart disease	Intake inversely associated with mortality from stroke (43)
Non-blinded randomized controlled trial conducted in ischemic stroke patients with low serum 25(OH)D levels	n/a	Vitamin D replenishment will improve the stroke outcome (75)
Randomized controlled trial in 73 patients assessing vitamin D & calcium on ischemic stroke outcomes	n/a	Benefit observed in ischemic stroke patients (76)
Serum 25-hydroxyvitamin D [25(OH)D] levels ≤ 20 ng/mL	Overweight African-Americans age 13–45 years	Arterial stiffness improved with vitamin D3 supplementation in vitamin D deficiency (78)
Serum 25-hydroxyvitamin D [25(OH)D] levels not measured in total of 2,690 patients who had a first episode of non-fatal ischemic stroke	Age 40–89 years old	Calcium supplementation along with vitamin D was not associated with an increased risk of ischemic stroke (89)

One crucial advantage of this review is that the population health studies examined encompass broad demographic diversity within the United States, as well as in Europe, Africa, China, and India for insights in global health, especially in developing, middle-income nations with growing stroke incidence. However, differences in environmental and socioeconomic conditions make comparisons and syntheses of international data difficult.

Furthermore, most research does not explore 1,25(OH)_2_D_3_'s neuroprotective pathways in depth. Additional research on 1,25(OH)_2_D_3_'s mechanisms could explain how 1,25(OH)_2_D_3_ mitigates the onset, severity, and functional outcomes of ischemic stroke. A causal relationship could be conclusively established if such mechanisms were fully explained. The various theories of neuroprotection may indicate which aspects of 1,25(OH)_2_D_3_'s physiological roles to examine, including growth factor expression, antithrombotic effects, and vasodilation to encourage blood flow. In addition, determining the mechanisms that explain the influence of variables such as sex and genetics would also be necessary for further investigation. The variable that has been least studied concerning stroke incidence is sun exposure. Much more research into stroke risk and 25(OH)D_3_ levels due to relative sun exposure (accounting for differences in geography, seasons, and other sub-factors) would offer significant clinical insight for stroke care and prevention in different regions.

Overall, this paper adds to the current knowledge of VD and ischemic stroke by synthesizing research on the relationship between 25(OH)D_3_ deficiency and stroke incidence, as well as the efficacy of supplementation for clinical treatment. Additional risk factors, including race, sex, and genetics, have all been incorporated into the analysis of stroke incidence, clarifying how such variables interact with 25(OH)D_3_D3 serum levels and contribute to stroke risk. Examining the results of the nascent research on supplementation may provide direction for future research.

## Conclusions and Future Direction

25(OH)D_3_ deficiency levels are a significant risk factor for ischemic stroke and may have prognostic value, especially for women and individuals with particular DBP SNPs. Worsening stroke severity and outcomes have also been associated with 25(OH)D_3_ deficiency (43–50). VD supplementation may reduce ischemic stroke risk and outcomes, but findings remain mixed, especially when considering the detrimental effects of high calcium supplementation. These findings highlight the need for randomized controlled trials to determine whether VD supplementation in patients with deficiency could be beneficial for the primary or secondary prevention of cardiovascular and cerebrovascular events. With high prevalence globally, VDD is not uncommon. It is associated with adverse health-related problems. Randomized controlled trials are therefore urgently needed to evaluate whether VD supplementation reduces the incidence of strokes and improves the outcome of poststroke patients.

## Author Contributions

KY proposed the topic of the review and provided guidance in the research process. NM reviewed the literature and drafted the paper with input and critical review from all authors. SD provided guidance in the research and writing process with suggestions on areas of investigation.

### Conflict of Interest

The authors declare that the research was conducted in the absence of any commercial or financial relationships that could be construed as a potential conflict of interest.

## Abbreviations

BBB: blood brain barrier
DBP: VD binding protein
HR: hazard ratio
IGF-1: Insulin-like growth factor 1
mRS: Modified rankin scale
NOS: nitric oxide synthase
RXR: retinoid X receptor
SDF1α: Stromal cell-derived factor 1α
SNP: Single nucleotide polymorphism (SNP)
VEGF: vascular endothelial growth factor
Vitamin D: VD
VDD: Vitamin D deficiency
VDRE: VD response elements.
